# The preference of onboard activities in a new age of automated driving

**DOI:** 10.1186/s12544-022-00540-7

**Published:** 2022-04-18

**Authors:** Jamil Hamadneh, Domokos Esztergár-Kiss

**Affiliations:** Faculty of Transportation Engineering and Vehicle Engineering, Budapest University of Technology and Economics (BME), Műegyetem rkp. 3, 1111 Budapest, Hungary

**Keywords:** Travel time, Multitasking, VOT, Autonomous vehicle, Onboard activities, Discrete choice modeling

## Abstract

According to the economic theory assumption, travelers tend to monetize travel time based on factors related to their individual and trip characteristics. In the literature, a limited number of studies evaluating onboard activities on traveler’s utility in the presence of the autonomous vehicle (AV) are found. In the current research, traveler preferences on board of three transport modes: individual-ride autonomous vehicle (IR-AV), shared-ride autonomous vehicle (SAV), and public transport (PT) are studied. The focus of this paper is the examination of travelers in urban areas, where traveling is relatively short, and the study of the travelers’ main trip purposes. The impact of travel time, travel cost, and main onboard activity is estimated based on a discrete choice experiment (DCE). The in-vehicle onboard activities are divided into six onboard activities, where active and passive activities are considered. An experimental design and a stated preference (SP) survey are carried out. The result of the SP survey is analyzed, where a Mixed Logit (ML) model, which includes various explanatory variables, is applied. The developed model contains such variables as trip time, trip cost, main onboard activity, frequent transport mode, job, age, and car ownership. These variables show various effects on the probability of choosing a transport mode. The impact of change in travel time, travel cost, and each of the six onboard activities on traveler preferences is highlighted. As a result, variations on the impact of time, cost, and onboard activities are demonstrated. Furthermore, it is presented that people prefer using IR-AV over SAV and PT, while the probability of choosing SAV is the lowest. Besides, reading and using social media affect the utility of travelers positively (i.e., higher probability) to a greater extent than other activities, while writing alone demonstrates negative utility.

## Introduction

The utilization of travel time on board of conventional transport modes is profoundly covered in the literature, where each transport mode impact has a certain level of utilization based on the traveler preferences, such as car-as-a-passenger travelers utilize their travel time more than the drivers. Generally, Guevara [21] states that travel time is divided into three parts, i.e., walking time, waiting time, and in-vehicle time. The in-vehicle time is the least unfavorable, while the waiting time is the most unfavorable [42]. The in-vehicle time is the longest part of the journey, which deserves more attention, and it is changeable with the presence of new transport modes or new vehicle designs. In the current research, the in-vehicle time is studied. People try to maximize their utility on board by conducting onboard activities, where some onboard activities can be done with the travelers’ carried tools. For example, tools include information and communication technology (ICT) tools, and onboard activities might be phone calls [24].

Belenky [5] shows that the selection of a transport mode is done based on the traveler preferences, where travelers try either to save time or to reduce the negative impact of the travel. People can switch to a transport mode to make the travel time more productive, for example, by conducting onboard activities [42]. It is found that those people who were born between 1980 and 2000 have more productive travel time than older generations because youngsters use the opportunity provided by the advancement of ICT [43]. Having ICT tools on board means higher chances of a pleasant journey. The availability of ICT tools on board a transport mode motivates travelers to conduct onboard activities, which has an impact on the travelers’ willingness to pay for saving travel time [67]. When examining traveling by trains, those travelers who are involved in onboard activities receive a lower value of travel time (VOT) than those who are not [4]. Furthermore, researchers find that as the comfort level on board a transport mode increases, the VOT decreases [32]. The environment on board of a transport mode, such as the noise, congestion, space, seat availability, makes an impact on conducting onboard activities [24]. On the other hand, the selection of a transport mode is affected by the traveler preferences, such preferences are a transport mode selection based on multitasking (i.e., conducting more than one activity onboard), and travelers sociodemographic variables [63]. Pawlak [52] prepares a systematic literature review demonstrating the effects of digital tools on travel time. The study includes solely digital tools and their impacts on travel time on board of conventional transport modes, while other portable tools that trigger travelers to conduct different activities are not shown. In addition, Pawlak’s focus is on conventional transport modes rather than autonomous vehicles (AVs).

In the literature, the conventional transport modes are the primary focus of research due to their availability on the market, while moderate is the attention to the travelers’ behavior on board of AVs. The fast development of technology makes the emergence of AVs, which might be operated in the near future, possible. AVs have different characteristics than conventional cars, for example, AVs provide a door-to-door service, convert drivers to passengers, and reduce the walking distance. These differences affect the travel behavior to a great extent [16]. The travelers’ behavior and preferences on board of various transport modes including AVs are studied by several researchers, such as Etzioni et al. [20], Pudāne et al. [55], Lee et al. [41], Polydoropoulou et al. [54] and Pudāne et al. [56]. However, these scholars pay little attention to mode choice in the automated driving age based on multitasking availability.

Etzioni et al. [20] study people’s acceptability of privately owned AVs across seven European countries, and they find large refrainment from the use of these new modes in all examined countries. The study does not differentiate between AVs and regular cars based on the utilization of in-vehicle time, it studies one type of AV uses (e.g., shared AVs are not mentioned), and the findings are built based on two variables: the travel time and the travel cost. Pudāne et al. [55] develop a model to optimize the use of travel time in the AV era. The model is built on the traveler preferences, where performing an activity on board gives a chance to make another one during the day. The study does not consider multitasking in detail and its connection to the mode choice, it focuses exclusively on the use of travel time on board of AVs. Moreover, Pudāne et al. [55] research does not study shared-ride autonomous vehicle (SAV). Lee et al. [41] examine the preference and heterogeneity of AVs in case of different travel distances. The study demonstrates the impact of travel distance on the transport mode selection considering car and AV. One of the findings presents that resting/sleeping and using ICT tools are the main activities that might be dominant on board of AV. However, the study does not differentiate between the various activities conducted by using ICT tools and their impacts on the choosing a car. Furthermore, according to the research, travelers choose a mode solely based on the travel time and travel cost rather than the possibility of onboard multitasking. Pudāne et al. [56] find that the readiness of travelers to multitask increases in case of AV as well as in case of users with higher education and income. The researchers study general activities rather than detailed ones, such as work activity, meal, sleep, and leisure activity. Milakis et al. [46] demonstrate heterogeneity in the acceptability of AV based on a panel of experts. The scholars focus on several attitudinal variables without mentioning multitasking as a factor in acceptability. Polydoropoulou et al. [54] highlight the differences between seven European countries based on the transport mode choice, where car, individual-ride autonomous vehicle (IR-AV), and SAV are examined. The authors find differences in the acceptance of a mode based on the companions’ gender. The study differentiates between the choices based on time, cost, and the companions’ gender rather than multitasking availability. AV is still not on the market,thus, further studies on the travelers’ potential behavior on board various types of AVs are needed, as well as evaluating the impact of onboard activities on the transport mode choice is necessary.

Previous studies analyze the in-vehicle travel behavior by considering different types/groups of onboard activities, where the conventional transport modes are the main focus. Moderate attention to multitasking can be found in the literature by considering the availability of AVs on the market. Furthermore, in previous studies, multitasking connected to AVs is not taken into consideration in more detail, and solely the general uses of multitasking options are studied. Besides, the trip purpose and the geographic area are not well-defined and covered in previous studies. The current research paper attempts to cover these gaps. Thus, the framework of this study is the analysis of travelers’ main trips in urban areas, which differs from the objectives of previous papers. Moreover, the selection of transport modes by the availability of main onboard activities is analyzed, where the preferences of travelers onboard are manifested. A detailed examination of main onboard activities based on the need for ICT tools is presented, where active and passive activities are demonstrated. The main contribution of this paper concentrates on estimating the utility of different onboard activities and their impact on the preferences of travelers’, when using the following transport modes: IR-AV, SAV, and public transport (PT). The selection of those modes because of their shared characteristics, such as no driver, and all people are converted to passengers. It is worth mentioning that IR-AV and SAV are assumed to provide a door-to-door service, and they are not owned by individuals.

The current paper is structured as follows: the introduction can be found in the first section, while the literature review, which summarizes the background and some relevant studies, is in the second section. Section 3 presents the methodology including the methods used in designing the survey and in the analysis. The following section demonstrates the results, while the discussion, where the findings are discussed, is presented in Sect. 5. Finally, the conclusion of the research is provided in Sect. 6.

## Literature review

According to the time allocation theory, travel time is considered a waste, and travelers plan their daily travels in a way to minimize this waste by involving themselves in onboard activities or by having a pleasant journey [24]. Jara-Díaz [32] states that travel time is seen as consumption, which means expense rather than the gaining of money. Travel time is utilized by travelers through conducting pleasant activities, such as using social media or sleeping [52]. Belenky [5] shows that people tend to pay money to compensate for the uncomfortable parts of their travel, for example, waiting, seating, and crowding, and to choose a suitable transport mode. The valuation of travel time is neither equal for all travelers nor constant with time due to the influence of such factors as the ability to multitask, the use of ICT tools, the trip purpose, the demographics, the transport mode, the travel condition, the geographic location, and the time of conducting the trip (e.g., season, working days, off days, and holidays) [5, 37, 65, 67].

Travel-based multitasking means that travelers plan their travel based on the possibility of conducting onboard activities, where ICT tools and vehicle automation are factors that facilitate multitasking on board [7]. The use of travel time is influenced by the selection of transport modes, where people choose transport modes based on the possible multitasking availability (i.e., conducting at least one onboard activity during the travel) [52]. Ettema and Verschuren [19] as well as Varghese and Jana [67] study the activities on board of conventional transport modes and the impacts of onboard activities on the VOT. Keseru et al. [34] show that the trip purpose has an effect on the use of ICT tools, which demonstrates the significance of onboard technology. Munkácsy et al. [49] demonstrate that talking to others is the prevailing activity on board of PT in Hungary. Furthermore, the use of ICT tools shows a decreasing tendency when the age of a traveler increases. Malokin et al. [44] use a revealed preference survey and highlight the modal shift based on multitasking, where the availability of ICT tools is an extremely important factor for travelers to conduct onboard activities, such as the availability of cellphone as a tool for making phone calls, and the availability of Internet provides an opportunity to use social media. Rhee et al. [58] and Mokhtarian et al. [47] assess the impact of onboard activities and ICT tools on the travelers. Rhee et al. [58] demonstrate the positive impact of onboard activities on the travelers, while Mokhtarian et al. [47] study the variables that make travelers feel tired, mentally/physically fatigued, or that the journey is un/pleasant during the travel. Pawlak [52] presents a systematic study that focuses on the impact of using digital tools on board on travel time. The study focuses on conventional transport modes, such as car, train, bus, and transit. The importance of digital tools in the perceived travel time is demonstrated in Pawlak’s study. Kyriakidis et al. [38] show that people might choose to use AV with the presence of human supervisor due to safety concerns.

Other researchers study and analyze travel behavior based on qualitative and quantitative variables that have a crucial impact on the transport mode choice models. Litman [42] shows that based on the qualitative measures, VOT reduction is less costly compared to infrastructure solutions (e.g., the construction of a new road to increase the speed). The researcher finds that people are capable to switch transport modes, if specific aspects are changed, such as an improvement in comfort or convenience, and service improvement in providing a suitable environment make travelers more productive and more attached to a particular transport mode. Belenky [5] highlights the importance of time utilization on board through multitasking, which minimizes the perceived travel time. Perk et al. [53] show that travel behavior differs from one person to another, and more interestingly, it often changes by time and by external factors even for the same person. Cirillo and Axhausen [15] state that travelers are willing to extend their travel when they find benefit or pleasure on board. Travelers’ characteristics impact the perceived travel time, for example, [36] find that people of high income and people with bad traveling conditions are more willing to pay for travel saving (i.e., to decrease the negativity of travel time) than children, retired, elderly, or unemployed travelers. Moreover, people try to minimize the travel time if the appropriate onboard environment, which provides the possibility to convert travel time into productive time by multitasking, is absent. Travelers choose faster transport modes to save time when the environment on board is not good enough for them to multitask. Thus, the saved time can be allocated to productive activities. Athira et al. [3] show that the saved time can be utilized by conducting additional onboard leisure activities. Ian Wallis Associates Ltd [30] presents different conclusions regarding the passengers and car drivers’ travel behavior depending on whether one or more passengers are in the car. The study demonstrates various findings, such as passengers can have more pleasant journeys than drivers or as the travel time and the number of companions increase the drivers acquire less pleasant journeys. The study demonstrates the impact of trip purpose on the importance of traveling, for example, commuting trips receive higher value than other trip types. These results demonstrate the variations between passengers and drivers. However, in the AV era, drivers are changed to passengers, which has an impact on travel behavior. Redmond and Mokhtarian [57] conclude that commuters are willing to reduce their commute time in case they travel more than their ideal commute time.

The rapid advancement of technology leads to the introduction of AVs on the market [35]. The evaluation of the impact of various subjective variables requires a discrete choice model based on those factors that affect the travel time from the travelers’ perspectives [32]. Several researchers perform discrete choice modeling and include AV in their models by using a stated preference (SP) survey. The properties of AV, which are different from that of the conventional cars, might gradually change the travel behavior assuming the people’s acceptability to use a transport mode driven by a machine [35, 63]. One of the travelers’ interests to switch to AVs is having more pleasant and productive time compared to other transport modes based on several factors, such as time, cost, and the possibility of conducting onboard activities [70]. If AVs provide higher utility for travelers than other transport modes, a desire might rise to use this new mode. A reduction in the travel time suggests an increase in the opportunity cost (i.e., the reduced time during the travel is transferred to the positive utility, which a traveler can conduct in another activity after finishing his/her journey), or the time spent on multitasking on board might reduce the time spent in activity thus providing an opportunity to be involved in other activities [23, 25, 29]. Pudāne et al. [55] state that conducting onboard activities in case of AV gives an opportunity for travelers to conduct other activities during the day, which increases the optimization of the travelers’ utility. Etzioni et al. [20] examine travelers’ acceptability of the privately owned AV and car in seven European countries, and the scholars find that people still stick to the car. Lee et al. [41] show the difference in using AV depending on the travel distance. The researchers study six types of multitasking such as resting/sleeping, using ICT tools, conducting business activities, eating lunch, reading a book or document, and others. One of the findings is that resting/sleeping and using ICT tools are the main activities that might be dominant on board of AV. Pudāne et al. [56] present that travelers with higher educational levels and incomes are more ready to multitask similarly the interest in multitasking increases when using AV. [46] demonstrate heterogeneity in the acceptability of AV based on a panel of experts. The researchers focus on several attitudinal variables regardless of the availability of multitasking as a factor for acceptability, where experts agree on some attitudinal variables and disagree with others, such as people are more likely to use AV in case of long distances. Polydoropoulou et al. [54] show the differences among seven European countries based on the transport mode choice, where private car, IR-AV, and SAV are the alternatives presented. The authors find differences in the acceptance of a mode based on the companions’ gender. Simoni et al. [62] find that people utilize their travel time on board of AV more than in case of other motorized transport modes. Gurumurthy and Kockelman [22] study AV and SAV concerning travel time, travel cost, and sociodemographic variables and conclude that AVs are more likely to be used for long travels. Lavieri and Bhat [40] study AV and SAV. The scholars state that the companion's type has an impact on the use of SAV, and the delay has more impact on the use of SAV than the companion. Regarding the specific user groups, Bozorg and Ali [9] show that high-income travelers get benefit from using AVs to a greater extent than other groups. Considering the different modes, Steck et al. [65] conclude that AV, whether it is shared or unshared, might reduce the VOT saving for people who make commuting trips. The authors find that the unshared version (i.e., AV) is more attractive than the shared version (i.e., SAV). Kolarova et al. [36] find that people are more likely to use AV compared to regular car, and people are more likely to use regular car than SAV. Other studies demonstrate an improvement in the utility of traveling when travelers conduct more onboard activities and are in a more comfortable environment without the stress and mental tension generated from driving [63, 68]. The vehicle design and the ease-of-use factors impact the travelers’ possibility to conduct onboard activities. A significant reduction in the disutility of traveling is obtained in long-distance traveling when people can engage in multitasking to a greater extent [63]. The onboard activities on AVs are different based on the trip purpose and the trip direction [68]. However, further empirical analysis is needed to evaluate the impact of AVs on travel behavior. It is worth mentioning that using IR-AV and SAV can provide a pleasant time to some groups of travelers as these modes remove the stress of driving and the transfer time, and increase the possibility of multitasking during traveling [31, 51].

The results of the previously discussed studies focus on the factors that impact the travelers’ behavior based on certain properties, such as trip purpose, transport mode, travel time, the role of the travelers (i.e., driver or passenger), and qualitative measures. Previous studies focus on the conventional transport modes and the use of ICT tools rather than on the types of AV and any portable tool on board, and no definition for the area of the study is given in the previous papers. A limited number of studies focus on multitasking on board of AV, and no studies that compare IR-AV, SAV, and PT based on multitasking are found. The gaps in previous studies have no connections to the trip purpose and the onboard activities. In the literature, scarcely can be found studies on the selection of a transport mode based on travel time, travel cost, and the availability of main types of onboard activities in IR-AV, SAV, and PT. Current research examines the travelers’ behavior while traveling to their main trip purposes in urban areas, where traveling is relatively short in which different presentations of onboard activities are used. As a result, the travelers’ preferences onboard and the factors that impact the potential selection of a transport mode in the AV era are discussed.

## Methodology

**Fig. 1:**
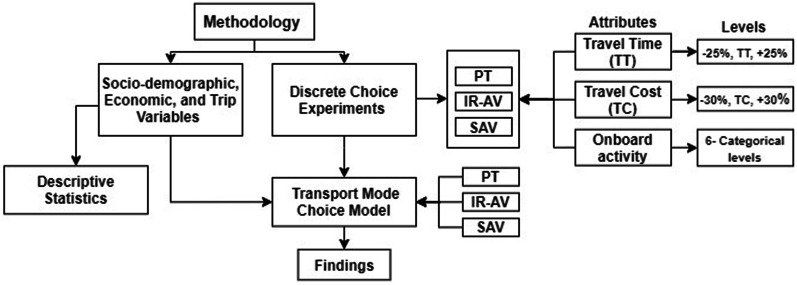
The methodological approach

The factors that have an impact on travel behavior are diverse, such as trip purpose, job, mode choice, cost, and other embedded variables like comfort, the internal design of the vehicle, and infrastructure. Onboard activities might affect travel behavior negatively or positively based on the traveler preferences. Figure 1 illustrates the methodological approach of the current paper.

Based on the literature, the most-used onboard activities in case of conventional transport modes are collected and reformulated to be used in this study. Additionally, the onboard activities are added to the characteristics of the alternatives in the discrete choice experiment (DCE) to estimate the utility of each onboard activity for every transport mode separately while developing a transport choice model. The transport mode choice model provides information on the added value (i.e., utility) of each attribute based on the travelers’ decisions. In addition, the preferences of travelers onboard are evaluated. In this research, a transport mode choice model concerning the travel time, travel cost, and main onboard activity is developed. The utility of the factors in the model including the availability of each onboard activity is estimated. Furthermore, the onboard activities are understood from the respondents’ perspective with respect to the chosen transport mode. This paper focuses on the in-vehicle time of IR-AV, SAV, and PT and presents how travelers select a transport mode based on their preferences and the characteristics of each alternative.

An SP survey is applied to study the travel behavior on board concerning main onboard activity, travel time, and travel cost. In the survey, sociodemographic and economic variables, trip characteristics, and a choice experiment are included. Furthermore, the discrete choice approach is used to study the travel behavior considering the IR-AV, SAV, and PT modes. The design for the DCE simulates the behavior of different types of travelers. The DCE contains three transport modes (i.e., alternatives) and three attributes with different levels, i.e., travel time, travel cost, and main onboard activity. Descriptive statistics are performed for the collected sample, which includes 525 respondents. The data present the respondents’ proportions of sociodemographic, economic, and trip variables such as age, gender, income, car ownership, education, employment, transport mode, trip purpose, and trip length.

In literature, there are several approaches that are used in studying the intention of travelers to use or adapt new technology, such as the Theory of Planned Behavior (TPB) [2], The Technological Acceptance Model (TAM) [14], The Unified Theory of Acceptance and Use of Technology (UTAUT) [48], and the Discrete choice Modeling [45]. The TPB has difficulties in conceptualizing and capturing the attitudes of users. The TAM approach is used to model how users accept a technological system [14]. The TAM is used mainly for educational purposes, and it is based on the ease of use and the benefits of individuals. The pitfalls of TAM are that it is not strong in prediction due to the limited explanatory and the lack of practical values. The UTAUT is widely applied in computer science. The UTAUT is a method that uses four key constructs to measure the acceptance of a new technology. The predominant modeling approach in transportation applications, which is widely used in developing the transport choice models, is the discrete choice modeling approach. The discrete choice modeling is applied to uncover how travelers value certain attributes of a product/service by asking travelers to select their choice over different hypothetical alternatives, such as transport modes and social sciences [45]. In current research, discrete choice modeling is selected. Discrete choice modeling is consistent with the random utility theory (i.e., the probability framework) [26]. The DCEs reflect the existing and proposed conditions (i.e., hypothetical market) of a problem, which enables the decision-makers to acquire rich data sources [39]. The DCE provides an evaluation for multiple options rather than one option, and it can give information on the willingness to pay for an individual characteristic of an option [39]. The data from the DCE are analyzed by using a discrete choice model. Various characteristics of the travelers are included in the model, too.

In this study, an SP survey method is used to extract responses that detect priorities, preferences, and the relative importance of individual features associated with the transport mode characteristics [26].

### DCE properties and specifications

The alternatives used in the DCEs are IR-AV, SAV, and PT. The attributes of time, the cost, and the main onboard activities are used with different levels for each, as shown in Table 1. The survey is distributed in Budapest, Hungary, and the cost of using conventional transport modes is obtained based on real data [10], while the cost of using IR-AV is taken from the literature [8]. The cost of using a personal car is set to 60 HUF per km (i.e., 0.175 Euro per km), and it is based on the average annual mileages traveled by people in Budapest and includes the parking fees per trip. On the other hand, the cost of using PT is 316 HUF per day (i.e., 0.93 Euro per km) based on the monthly PT pass (i.e., 9500 HUF/27.9 Euro), and 20 working-days of traveling are considered per month [17, 66]. To determine the average trip distance and trip time for the travelers in the urban area of Budapest, the simulations based on the household survey data conducted by using MATSim open-source software are taken. Around four km average trip distance and 30 min average trip time are defined [23]. In the survey, travelers are asked to select one alternative from a choice set in a block based on their preferences and the information available in the choice set. The levels are selected for each attribute to ensure that travelers can have a trade-off among the alternatives.

**Table 1:** DCE attributes and levels

Levels	Attributes		
	Main onboard activities	Total trip cost (HUF)*	Total trip time (minute)
L 1	Reading	1.30*(TC)	1.25*(TT)
L 2	Writing	TC	TT
L 3	Talking	0.70*(TC)	0.75*(TT)
L 4	Using social media and gaming		
L 5	Eating and drinking		
L 6	Others		

1Euro = 340HUF

Furthermore, the main onboard activities are defined as the possibility to conduct onboard activities, such as reading, writing, using social media, talking, or eating/drinking. Six onboard activities are formulated, i.e., (1) reading, (2) writing, (3) talking, (4) using social media and gaming, (5) eating/drinking, and (6) doing nothing on board (i.e., doing others like window gazing, sleeping, etc.). In the case of reading or writing, one can use either paper-based (e.g., book or sheet) or technological tools (e.g., mobile, laptop, or tablet) during the travel. Talking with strangers, family, acquaintances, or friends is conducted either face to face or by using technological tools inside the vehicle. Facebook, YouTube, Twitter, Instagram are examples of social media. The sixth activity (i.e., doing nothing) means that travelers do not conduct any activity of the previous six activities (however, they have feelings: they are bored, anxious, unpleasant, stressed, etc.). The aim of this methodology is to estimate the utility of an activity on the transport mode when a traveler chooses one of the sixth activities as a main activity with the possibility to conduct other activities as secondary activities. Decision-makers can use the output of this analysis as an input to improve the transport system.

Table 1 presents the options in case of each transport mode based on the six onboard activities. Moreover, onboard activity has a contribution to mode choice, for example, reading and writing for work purposes is different than reading and writing for leisure as the requirements of these onboard activities (e.g., privacy) are different. Since not all activities can be conducted efficiently on all transport modes, travelers choose a transport mode over the others to be able to conduct a certain activity, while other activities are considered secondary and have little effect on the travelers’ utility. The definition of onboard activities provides information to understand the utility of different onboard activities that form the main concern of travelers. Thus, travelers are asked to select IR-AV, SAV, or PT after considering the time, the cost, and the possible main onboard activities considering the possibility of conducting other secondary activities based on traveler’s preferences. As a summary, Table 1 illustrates the attributes and levels used in generating the choice sets. The attributes’ levels are distributed randomly in the choice experiments.

An example of choice combination from the survey is shown in Table 2. For example, a traveler who chooses IR-AV to travel to his/her main activity means that his/her preference is conducting mainly talking activity onboard where trip time 20 min and trip cost 175 HUF is given. The preferred onboard activity of a traveler is talking activity based on his personal preferences and trip characteristics. Thus, the traveler’s preference of onboard activities affects the choice of a transport mode.

**Table 2:** One choice combination

			
Attributes	IR-AV	SAV	PT
Total trip cost (HUF)	175	325	175
Total trip time (minute)	20	30	20
Availability of main onboard activity	Talking	Reading	Writing

### The DCE design methods

The DCE design is constructed by using a fractional factorial design [59]. The attributes of the DCEs are uncorrelated, and the alternatives are mutually exclusive. The respondents answer six questions. The number of levels in each block is equal, and the number of choice sets in each block is equal. Each choice set has three alternatives, each alternative has its level values (i.e., constraints), each alternative has the same number of attributes, and the logical choice sets are solely designed. The orthogonal design, balance, overlap, full factorial, and fractional factorial design, efficient design, choice sets, and blocks are examined to ensure the adequate design of the DCE [39]. Orthogonal design is applied to analyze the correlation among the attributes and to assure that all attributes are independent.

The number of the generated scenarios of the full factorial design is large, so the fractional factorial design, which includes the selected combinations of alternatives, attributes, and levels, is applied [12]. The fractional factorial design is realized by using RStudio to find the possible combinations. The choice set creation is conducted by using simultaneous choice sets [13, 60]. Practically, a block is randomly given to a respondent, so instead of all choice sets, the questions in the given block are answered [33]. The “Lma.design” function from the “support.CEs” package is used to create the DCEs from the orthogonal main-effect array, where each row represents a combination set. The results are 72 choice sets/combinations distributed equally to 12 blocks [1]. The required sample size for conducting the analysis is 330 respondents (500 *(the largest number of levels) / (the number of scenarios * the number of attributes)) based on de Bekker-Grob et al. [18].

It is worth mentioning that Stata Software 16.1 is used to develop the DCE and to analyze the collected data (Stata [64].

The choice sets are distributed randomly for participants where each participant answers one random block (i.e., choice set). Moreover, the attributes are distributed and equally randomly inside the block, as mentioned by Lancsar and Louviere [39].

### Random utility theory

In a traveler choice context, the random utility theory assumes that every individual is a rational decision-maker, and travelers try to maximize the utility based on their choices [11]. Certainty in stating which alternative (i.e., transport mode) a traveler selects is not applicable, but the probability that a traveler chooses one alternative over the others is possible to be estimated. This can be expressed by Eq. (1).

1 $${P({\mathrm{c}}_{j}/\mathrm{C}) =\mathrm{P}(U}_{{\mathrm{c}}_{j}}>{U}_{k}) \forall k\ne j, k\in C$$

where P (c_j_/C) is the probability of an individual to choose alternative (j) from choice set (c), C is the available choice sets for a traveler, U_cj_ is the perceived utility of choosing alternative (j) from choice set c, where the obtained utility is larger than the utility of all other alternatives (k) in choice set (c) [11]. The utility function consists of two parts, a deterministic and a stochastic part, as shown in Eq. (2).

2 $${U}_{j}={V}_{j}+{\upvarepsilon }_{j}$$

where V is the deterministic part, and ε is the stochastic part. The deterministic part represents the travelers’ mean perceived utility when choosing an alternative [11]. The random part represents the unknown deviation of the travelers’ utility from the mean value, and it captures the uncertainty in the choice modeling [11]. The characteristics of the stochastic part determine the type of model that fits the data and makes the produced model more accurate.

### Mixed Logit (ML) model

The Mixed Logit (ML) model does not require the independence of irrelevant alternatives (IIA) for the stochastic part as in the Multinomial Logit model, which overcomes the taste variation issues (i.e., the marginal utility is random ($\beta$)). The utility of individual (i), who chooses alternative (j), in choice situation (c) is given in Eq. (3).

3 $${U}_{ijc}={V({\beta }_{i}/X}_{nijc})+{\upvarepsilon }_{ijc}$$

where ${X}_{nijc}$ is the vector of the observed independent variables (n), including the attributes of the alternatives and the travelers’ socio-demographic characteristics, which is an important part of the choice selection. ${\upvarepsilon }_{ijc}$ is a random error, independent and identically distributed (IID) extreme value type 1 (i.e., Gumbel distribution) across all alternatives, individuals, and choice situations. Both ${\beta }_{i}$ and ${\upvarepsilon }_{ijc}$ are treated as stochastic parameters, which influence the accuracy of the model because they are not observed directly [27]. The IID is restrictive as it does not allow the alternatives to be correlated across each other because of the information which might be the cause and unseen to the analyst. Therefore, the stochastic component is divided into two parts. One part is IID (${\upvarepsilon }_{ijc})$ and the other part (${\upeta }_{ijc})$ is correlated over the alternatives and heteroskedastic, as shown in Eq. (4).

4 $${U}_{ijc}={V({\beta }_{i}/X}_{nijc})+{\upeta }_{ijc}+{\upvarepsilon }_{ijc}$$

where $${\upeta }_{ijc}$$ is a random term with zero mean property, whose distribution over the individuals and the alternatives depends in general on the underlying parameters and the observed data relating to alternatives (j) and individuals (i); and ε_iq_ is a random term with zero means, which is IID over the alternatives and does not depend on the underlying parameters or data. The ML class of the models assumes a general distribution for η and an IID extreme value type 1 distribution for ε. That is, η can take on several distributional forms, such as normal, uniform, lognormal, and triangular [28]. The ML model is the integral of the standard logit probabilities over a density of parameters; Eq. (5) shows the general form of the utility function of the ML model. The unconditional probability ($${P}_{i})$$ of the choice sequence, which is estimated by maximum likelihood estimation is the following.

5 $${P}_{i}=\int {L}_{i}\left(\upbeta \right) f\left(\beta \right)d(\beta )$$

where ${L}_{i}$ stands for the logit probability at β, and $f\left(\beta \right)$ is a density function. The probability that individual (i) selects alternative (j) from a set of k alternatives in time c, is conditional on β, as in Eq. (6).

6 $${L}_{ijc}=\left(\frac{\mathrm{exp}({X}_{ijc}{\beta }_{i})}{\sum_{j=1}^{K}\mathrm{exp}({X}_{ijc}{\beta }_{i})}\right)$$

The panel data-mixed logit model, which is known as the heteroskedastic error component type model, is used in this paper to capture the unobserved factors and the correlations between the choices selected by the same participant (i.e., they are shown in the alternative constant) [6].

### Model specifications and design

The utility function for choosing one transport mode over others is studied for three factors, i.e., travel time, travel cost, and main onboard activity. The utility function of the model is given by Eq. (7).

7 $$\begin{aligned} U_{ijc } & = \beta_{o \left( i \right)} + \beta_{TC \left( i \right)} *TC + \beta_{ TT \left( i \right)} * TT + \beta_{ OA \left( i \right)} * OA \left( {Dummy} \right) + \beta_{Tranport\,mode \left( i \right)} \\ & \quad *The\, regular\,used\,tranport\,mode\, \left( {Dummy} \right)*Dummy_{j} + \beta_{Age \left( i \right)} \\ & \quad *Age \left( {Dummy} \right)*Dummy_{j} + \beta_{Job \left( i \right)} \\ & \quad *Job \left( {Dummy} \right)*Dummy_{j} + \beta_{car\,ownership\left( i \right)} \\ & \quad *car\,ownership \left( {Dummy} \right)*Dummy_{j} + (\upvarepsilon + \upeta )_{ijc } \\ \end{aligned}$$

where ${U}_{ijc}$ is the utility of alternative (j) selected by individual (i) at time c, TC is the travel cost, TT is the travel time, and OA represents the main onboard activity. ${\beta }_{o (i)}{, \beta }_{TC \left(i\right)},,{\beta }_{ ML \left(i\right)}, { \beta }_{Tranport mode \left(i\right)}, {\beta }_{Age \left(i\right)},{ \beta }_{Job \left(i\right)},{ and \beta }_{car ownership\left(i\right)}$ are parameters of the observed variables to be estimated from the collected data, and $\upvarepsilon +\upeta$ represents an indeterministic error. Each case specific variable is multiplied by the dummy of alternative. It is worth mentioning that the regular transport mode stands for the transport mode used by the respondents to reach their main trip purpose. The VOT is given in Eq. (8).

8 $$VOT=\frac{{\beta }_{tt}}{{\beta }_{tc}}.$$

## Results

### The description of the sample

The SP survey is distributed during March and April 2020 in Budapest, Hungary by using the LimeSurvey tool [61]. The survey is sent to people via social media platforms and emails supported by an illustration and text. The impact of COVID-19 is not considered because participants are asked to answer based on their normal, pre-pandemic situation. The sample data includes 525 participants, who record their sociodemographic and travel characteristics, as shown in Table 3. The composition of the sample data is as follows. Most of the participants (84.76%) have undergraduate/graduate certificates. The results demonstrate the respondents’ educational level, which might reveal the quality of the answers and the degree of understanding in the survey. In the study, the representation of gender is reflected. The sample presents an almost equal representation of males and females. The representation of people who own a personal car is 34.67%. People with different income levels are reported in the table, where the majority with 38.10% is in the low-income class (i.e., less than 650 Euro per month), while 25.52% have middle income (i.e., more than 650 Euro but less than 1250 Euro), and 14.67% report high income (i.e., more than 1250 Euro). The age categories show that 56.19% of the participants are between 25 and 54 years old, while 37.9% is the percentage of the respondents in the 15–24 age category, and low percentages are found for other groups. The conclusion from the age statistics is that the study primarily examines the preferences of young and middle-aged people; thus, those who are between 15 and 54. The occupation categories make an impact on the traveler preferences; the survey collects eight types of jobs, as shown in Table 3. Around 43.62% of the participants are full-time workers, while 7.05% of the respondents are part-time workers, the percentages of students are 37.33%, and other groups are represented by low percentages. It is worth mentioning that the survey is distributed among different types of user groups but primarily among university students. The sample composition is different than the population due to the sensitivity of the result, the selection of respondents by email based on educational level is done. It is worth mentioning that the survey ensures the ethics of research, such as confidentiality, anonymity, and other issues. Thus, due to the applied channels to distribute the surveys, the age group of 15–24 does not include people less than 18 years old (i.e., this group includes bachelor students and master students). The range of the group (i.e., 15–24) is kept for standardization.

**Table 3:** Descriptive statistics on the sociodemographic variables

Category	%	Category	%	Category	%
Educational level		Income		Employment	
High school	11.24	Low	38.10	Workers	50.67
Undergraduate studies	53.90	Medium	25.52	Student	37.34
Graduate studies	30.86	High	14.67	Unemployed	4.38
Others	4.00	No answer	21.71	Homemaker	4.00
Gender		Age		Self-employed	1.52
Female	48.57	15–24	37.90	Retired	1.71
Male	51.43	25–54	56.19	Others	0.38
Car ownership		55–64	4.76		
Yes	34.67	+ 65	1.15		
No	65.33				

The participants record their travel characteristics such as transport mode, trip purpose, and travel time, as shown in Table 4. Most of the sample (41.71%) use PT, around 17.9% of the respondents drive their personal cars, and 11.4% use non-motorized modes. The number of participants who go to work and for educational trips is the highest with 50.1% and 37.52%, respectively. Furthermore, the leisure and other trip purposes are indicated by 3.24% of the participants. The travel time to the main destinations is recorded, where more than 70% travel less than 30 min. It is concluded that the majority travel from 10 to 30 min. The short travel time indicates that participants conduct trips within urban areas, especially when they go to educational institutes or workplaces.

**Table 4:** Participants’ trip purposes, transport modes, and trip time statistics

Main daily transport mode	%	Main daily trip purpose	%	Trip time	%
Car as a driver	17.90	Work	50.10	< 10 min	16.63
Car as a passenger	23.81	Shopping	2.86	> 10 min and < 20 min	25.81
Taxi	4.38	Education	37.51	> 20 min and < 30 min	20.27
PT	41.71	Home	6.29	> 30 min and < 40 min	13.01
Bicycle	3.24	Leisure or others	3.24	> 40 min and < 50 min	8.22
Walking	8.01			> 50 min and < 60 min	6.50
Others	0.95			> 60 min	9.56

### Model development

The effects of some socio-demographic, economic, and trip variables on the potential mode choice are demonstrated. The results of the SP survey, which includes the preferences of 525 people in Budapest, Hungary, toward three transport modes (i.e., IR-AV, SAV, and PT), are analyzed by Stata software 16.1. The ML model is applied, and the transport mode choice model including the travel time, travel cost, onboard activity, transport mode, age, job, and car ownership variables (i.e., some insignificant parameters are kept for comparison) is developed. It is essential to know that the superior model is selected from the different forms of examined models, such as random, log normal, uniform, triangle, and the gaussian tested distributions of the errors in the developed model. The utility of the various variables on the potential transport mode choice is explained and illustrated in tables and graphs to see the relationship of the variables.

### Model fitting information

The panel data-mixed logit model is developed to simulate the travelers’ behavior when choosing transport modes by concerning the travel time, travel cost, and six onboard activities, as shown in Table 5. The model with the lowest Bayesian Information Criterion (BIC) is selected. The statistics of the model are reported at the bottom of the table, where the model is statistically significant with 9450 observations, the log simulated likelihood is − 3382.556, and the BIC is 6837.609. The insignificant parameters are kept in the model for comparison. It is worth mentioning that travelers choose transport modes based on the DCE. Doing secondary activities is not studied here because travelers can conduct more than one activity at the same time, such as using social media and eating.

**Table 5:** Panel data—mixed multinomial logistic regression

Alternative	Attribute		Coefficient value (β)	Std. Error	z	*P* > z	Exp(B)
	Trip time	Time	− 0.029	0.005	− 5.270	0.000*	0.97
	Trip cost	Cost	− 0.002	0.0003	− 6.670	0.000*	1.00
	Onboard activity	Reading	0.268	0.077	3.490	0.000*	1.31
		Writing	− 0.111	0.082	− 1.211	0.117	0.89
		Talking	0.270	0.077	3.500	0.000*	1.31
		Using social media and gaming	0.311	0.077	4.050	0.000*	1.36
		Eating/drinking	0.077	0.080	1.875	0.031**	1.08
		Doing nothing/others	Base				
SAV	The regular transport mode	Car as a driver	Base				
		Car as a passenger	0.509	0.155	3.280	0.001*	1.66
		Taxi	0.207	0.282	0.730	0.463	1.23
		PT	0.278	0.173	1.610	0.10***	1.32
		Bicycle	0.091	0.010	0.930	0.352	1.10
		Walking	0.436	0.217	2.010	0.044**	1.55
		Others	0.009	0.396	1.20	0.235	1.01
	Age	15–24	Base				
		25–54	− 0.502	0.195	− 2.570	0.010**	0.61
		55–64	− 0.382	0.419	− 1.385	0.166	0.68
		+ 65	0.466	0.630	0.740	0.460	1.59
	Job	Full-time worker	Base				
		Part-time worker	− 0.006	0.181	− 0.630	0.529	0.99
		Student	− 0.333	0.134	− 2.470	0.013**	0.72
		Homemaker	− 0.356	0.226	− 1.580	0.115	0.70
		Unemployed	− 0.128	0.219	− 0.850	0.395	0.88
		Self-employed	0.021	0.425	0.650	0.514	1.02
		Retired	− 0.813	0.395	− 2.060	0.039**	0.44
		Others	0.725	0.649	1.420	0.156	2.06
	Car ownership	Yes	Base				
		No	0.258	0.201	1.280	0.200	1.29
PT	The regular transport mode	Car as a driver	Base				
		Car as a passenger	− 0.036	0.149	− 0.620	0.532	0.96
		Taxi	0.319	0.256	1.240	0.213	1.38
		PT	− 0.324	0.168	− 1.930	0.053***	0.72
		Bicycle	− 0.293	0.290	− 1.010	0.313	0.75
		Walking	− 0.152	0.215	− 0.971	0.331	0.86
		Others	− 1.393	0.522	− 2.670	0.008*	0.25
	Age	15–24	Base				
		25–54	− 0.281	0.210	− 1.340	0.181	0.76
		55–64	− 0.205	0.386	− 0.872	0.383	0.81
		+ 65	2.241	0.874	2.570	0.010**	9.40
	Job	Full-time worker	Base				
		Part-time worker	− 0.404	0.195	− 2.070	0.039**	0.67
		Student	− 0.264	0.136	− 1.940	0.052***	0.77
		Homemaker	− 0.727	0.245	− 2.970	0.003*	0.48
		Unemployed	− 0.424	0.135	− 1.800	0.072***	0.65
		Self-employed	0.723	0.262	2.000	0.046**	2.06
		Retired	− 2.492	0.359	− 4.460	0.000*	0.08
		Others	− 1.274	0.325	− 1.340	0.180	0.28
	Car ownership	Yes	Base				
		No	0.243	0.144	1.501	0.133	1.28
SAV		β_0_	− 0.145	0.202	− 0.720	0.471	0.87
PT		β_0_	0.217	0.199	1.090	0.277	1.24
IR-AV		Base					
The number of observations = 9450							
Chi2(2) = 330.29							
Log simulated-likelihood = − 3310.88							
Prob > chi2 = 0.000							
AIC = 6705.771							
BIC = 6960.088							

*p < 0.01; **p < 0.05; ***p < 0.1

### Model estimates

It is shown in the results that both the travel time and travel cost have a negative sign, which demonstrates that the travelers’ decisions are affected negatively by increasing the time and cost during the travel; on the other hand, onboard activity has varied effects (i.e., positive and negative) on the transport mode choice. A one-unit increase in the time variable is associated with a 0.029 decrease in choosing a transport mode, while a one-unit increase in the cost variable is associated with a 0.002 decrease in choosing a transport mode. It has to be noted that the model is developed based on the trip cost of the HUF unit and the trip time of the minute unit. From the marginal effects, the travelers’ willingness to pay for saving travel time is estimated by using Eq. (8). Based on the statistical results of the developed model, the travelers’ VOT is 870 HUF per hour (i.e., 2.64 Euro per hour).

In the developed model, doing nothing/others is used as a reference category. Furthermore, all variables are significant at a confidence level of 95% except for writing activity, which shows a significant result at a confidence level of 85%. Reading activity on board shows a positive value, which leads to the probability of selecting a transport mode with a reading possibility to be 1.31 larger than the probability of doing nothing (i.e., the relative risk ratio). Writing activity on board shows a negative sign, which means that the probability of selecting a transport mode with the possibility of onboard writing is affected negatively compared to the reference category. Thus, writing on vehicles is generally not preferred during the travel; the probability of writing on board is lower by 0.89 than the probability of the doing nothing activity. Talking activity on board has a higher probability than the probability of doing nothing (i.e., the relative risk ratio is 1.31), and the activity is significant in the model. The using social media and gaming activities demonstrate a 1.36 higher probability than the probability of doing nothing. Eating/drinking shows a low positive value, which means that the probability of using a transport mode with eating/drinking possibility is 1.08 higher than the probability of doing nothing.

Moreover, other variables, such as transport mode, trip purpose, age, job, and car ownership, are examined in the model to assess their effects on the travelers’ utility. The coefficient value (β) represents the utility while the Exp (β) represents the probability ratio. The significance of these variables is varied, as shown in Table 5.

### For the regular transport mode variable

In case of SAV, the car as a passenger, PT, and walking show significant results at a confidence level of 90%. The relative risk ratio switching from the car as a driver to the car as a passenger for being in SAV is 1.66 (i.e., the probability of staying in SAV for travelers who use car as a passenger is higher than who use car as a driver), the relative risk ratio switching from the car as a driver to walking for being in SAV is 1.55, and the relative risk ratio switching from the car as a driver to PT for being in SAV increases is 1.32. It is demonstrated that SAV is preferred by car as a passenger, walking, and PT users over the car as a driver based on the observed variable transport mode. While other insignificant variables are explained for comparisons, such as the relative risk ratio switching from the car as a driver to taxi for being in SAV 1.23, the relative risk ratio switching from the car as a driver to bicycle for being in SAV 1.1, and the relative risk ratio switching from the car as a driver to others for being in SAV 1.01. The insignificant results of those transport modes are potentially associated with the low proportions. The least probability to use SAV is for drivers (i.e., car as a driver) because all other transport modes show positive values.

In case of PT, PT and “others” modes show significant results at a confidence level of 90%. The findings present that the relative risk ratio switching from the car as a driver to PT for being in PT is 0.72 (i.e., the probability of staying in PT for travelers who use PT is lower than who use car as a driver), and the relative risk ratio switching from the car as a driver to others for being in PT is 0.25. It is demonstrated that travelers PT is preferred by the car as a driver over PT and others, based on the observed transport modes. While other insignificant variables are explained for comparison, such as the relative risk ratio switching from the car as a driver to the car as a passenger for being in PT is 0.96, the relative risk ratio switching from the car as a driver to taxi for being in PT is 1.38, the relative risk ratio switching from the car as a driver to bicycle for being in PT is 0.75, the relative risk ratio switching from the car as a driver to walking for being in PT is 0.86. The highest probability to use PT is for drivers (i.e., car as a driver) because all other transport modes show negative values except for the insignificant result of taxi, while PT is better environment for conducting main onboard activities over regular cars.

### For the age groups variable

In case of SAV, solely the age group of 25–54 is a significant result at a confidence level of 95%. The relative risk ratio moving from the age group of 15–24 to the age group of 25–54 for being in SAV is 0.61 (i.e., the probability of staying in SAV for travelers whose ages are 25–54 is lower than those travelers’ whose ages are 15–24). Other groups show insignificant results, such as the relative risk ratio moving from the age group of 15–24 to the age group of 55–64 for being in SAV is 0.68, and the relative risk ratio moving from the age group of 15–24 to the age group of 65 + for being in SAV is 1.59.

In case of PT, solely the 65 + group is significant, where the relative risk ratio moving from the age group of 15–24 to the age group of 65 + for being in PT is 9.4 (i.e., the probability of staying in PT for travelers whose ages are 65 + is higher than those travelers ages are 15–24). The age group of 25–54 and 55–64 show insignificant results, and the findings show that the relative risk ratios moving from the age group of 15–24 to the age group of 25–54 and 55–64 for being in PT are 0.76 and 0.81, respectively. As a result, the travelers whose ages are 25–54 are more likely to use SAV and PT than other age groups.

### For the job type variable

The job type contributes to the travel behavior, where choosing a transport mode is affected by the observed variable.

In case of SAV, the retired group and students show significant results, while other job types show insignificant results. The significant results are explained, such as the relative risk ratio moving from the full-time worker group to students for being in SAV is 0.72 (i.e., the probability of staying in SAV for travelers who are students is lower than those who are full-time workers), and the relative risk ratio moving from the full-time worker group to the retired group for being in SAV is 0.44. Thus, full-time workers are more likely to use SAV than students and retired people.

In case of PT, the relative risk ratio moving from the full-time worker group to part-time workers for being in PT is 0.67 (i.e., the probability of staying in PT for travelers who are part-time workers is lower than who are full-time workers), the relative risk ratio moving from the full-time workers to students for being in PT is 0.77, the relative risk ratio moving from the full-time worker group to homemakers for being in PT is 0.48, the relative risk ratio moving from the full-time workers to unemployed for being in PT is 0.65, the relative risk ratio moving from the full-time worker group to self-employed for being in PT is 2.06, the relative risk ratio moving from the full-time workers to the retired group for being in PT is 0.08, and the relative moving from the full-time worker group to others for being in PT is 0.28. All job types show significant results except for others. Thus, full-time workers are more likely to use PT by all job types except others.

### Car ownership variable

Additionally, car ownership affects the travelers’ decisions when they choose a transport mode.

In case of SAV, the findings demonstrate that travelers owning private cars are less willing to choose SAV. The relative risk ratio moving from car owners to those who do not own a car for being in SAV is 1.29.

In case of PT, the relative risk ratio moving from car ownership to those who do not own a car for being in PT is 1.28. Thus, having a car increases the probability of avoiding PT.

The alternative specific constant (β_0_) presents the relative risk ratio of choosing one alternative without considering the observed variables, where the relative risk ratio to use SAV over IR-AV is 0.87, and the relative risk ratio to use PT over IR-AV is 1.24. Thus, travelers are more willing to use PT than IR-AV, while they are more ready to use IR-AV than SAV.

### Margins

**Fig. 2:**
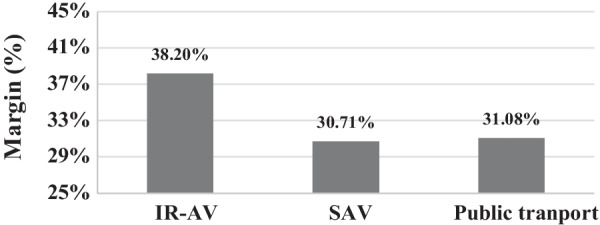
The predictive margins with 95% confidence interval

The predictive margins of the model show that the expected probability to choose IR-AV is 38.2%, to choose SAV is 30.7%, and to choose PT is 31.1% at a confidence level of 95%, as shown in Fig. 2.

**Fig. 3:**
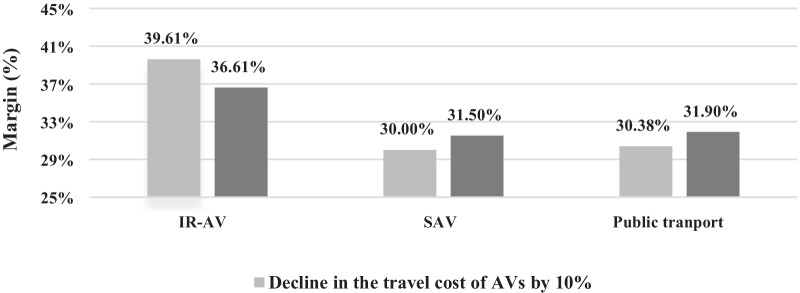
The predictive margins with 95% confidence level at 10% increment in the trip time of IR-AV and 10% decline in the trip cost of IR-AV

From Table 5, the probability of choosing specific alternative changes if one of the observed variables is changed, such as modifying the travel cost of a specific alternative to know the effects on the probability of choosing any of the other alternatives is estimated. The changes in travel time, travel cost and main onboard activity are discussed. Figure 3 shows that the change in trip time by 10% in IR-AV lead to a change in the margins of IR-AV, SAV, and PT. Furthermore, a reduction in the trip cost by 10% leads to a change in the margins of IR-AV, SAV, and PT, as shown in Fig. 3. As a result of the companions, it is found that the margins increase when the trip cost is decreased, while a decrease in the margins occurs when the trip time is increased for the IR-AV alternative.

**Fig. 4:**
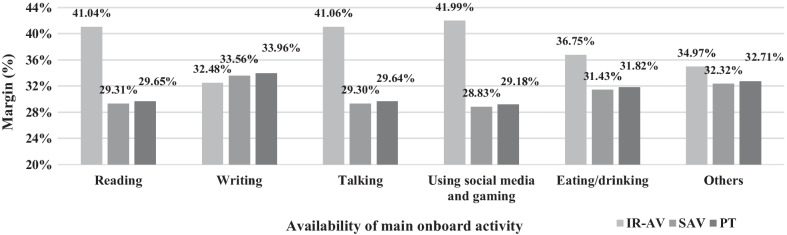
The predictive margins when the main onboard activity is possible on board of IR-AV

**Fig. 5:**
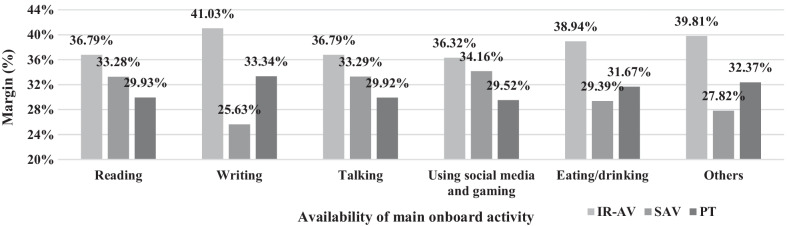
The predictive margins when the main onboard activity is possible on board of SAV

**Fig. 6:**
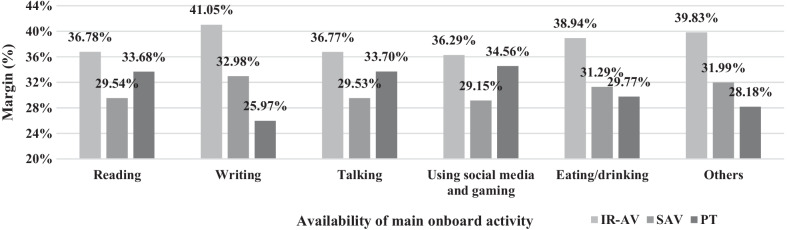
The predictive margins when the main onboard activity is possible on board of PT

Furthermore, the change in the margins across main onboard activity possibility is demonstrated in Figs. 4, 5 and 6. The margins are significant at confidence level of 95% with a standard error of less than 2%. The probability of an individual choosing a transport mode is varied across the availability of main onboard activities. Figure 4 shows the changes in margins when onboard activities are altered in IR-AV. For example, the margins of IR-AV, SAV, and PT when a traveler mainly conducts reading activity onboard is 41.04%, 29.31%, and 29.65%, respectively. This margin is produced based on the change in the attributes of IR-AV while the opponents transport modes attributes are held constant. The margins show that IR-AV has the largest margin across all onboard activities.

Figure 5 shows the changes in margins when onboard activities are altered in SAV. For example, the margins of IR-AV, SAV, and PT when a traveler mainly conducts reading activity onboard is 36.79%, 33.28%, and 29.93%, respectively. This margin is produced based on the change in the attributes of SAV while the opponents' transport modes attributes are held constant. It is shown that IR-AV has the highest margin across all onboard activities, while the margins of SAV across onboard activities show higher and lower margins compared to the PT. For example, the margins of SAV are higher than the margins of PT, when reading, talking, and using social media and gaming activities are the main onboard activity.

Figure 6 shows the changes in margins when onboard activities are altered in PT. For example, the margins of IR-AV are still higher than the margins of SAV and PT. This margin is produced based on the change in the attributes of PT while the opponents' transport modes attributes are held constant. The margins of PT across onboard activities show higher and lower margins compared to the SAV. For example, the margins of PT are higher than the margins of SAV, when reading, talking, and using social media and gaming activities are the main onboard activity.

In summary, finding the margin of changing attributes in each transport mode with respect to conducting an onboard activity is illustrated in previous figures (Figs. 4, 5, and 6). The impact of changing main onboard activities on the margin of a selected transport mode (i.e., IR-AV, SAV, and PT) is evaluated, where a traveler chooses IR-AV, SAV, or PT, then he/she chooses a main onboard activity based on his/her preferences (i.e., a traveler changes the main onboard activity on his/her selected transport mode.” Figures 4, 5, and 6 shows the margin of each transport mode across main onboard activity possibilities in IR-AV, SAV, and PT. For example, the margin of IR-AV when a traveler mainly conducts reading activity onboard is 41.01%, where a change in the attribute of main onboard activity of IR-AV is occurred (i.e., a traveler chooses IR-AV and he/she make changes on the possible main onboard activities). The margin of SAV when a traveler mainly conducts reading activity onboard is 33.27% where a change in the attribute of main onboard activity of SAV is occurred. The margin of PT when a traveler mainly conducts reading activity onboard is 33.62% where a change in the attribute of main onboard activity of PT is occurred. These margins are produced based on the change on the attributes of solely alternative while the opponents transport modes attributes are held constant. Figures 4, 5, and 6 is derived from the previous three figures.

From the margins in the figures above, the IR-AV is superior which means people are more likely to choose IR-AV compared to PT and SAV, while the PT and SAV show slight margin differences. Moreover, the impact of changing on the main onboard activity of a transport mode is varied, for example, the changes in the IR-AV leads to 33.56% in case of SAV while the changes in the SAV leads to 25.63% predicative margin. Thus, the availability of main activity on board of a transport mode affect the predication.

**Fig. 7:**
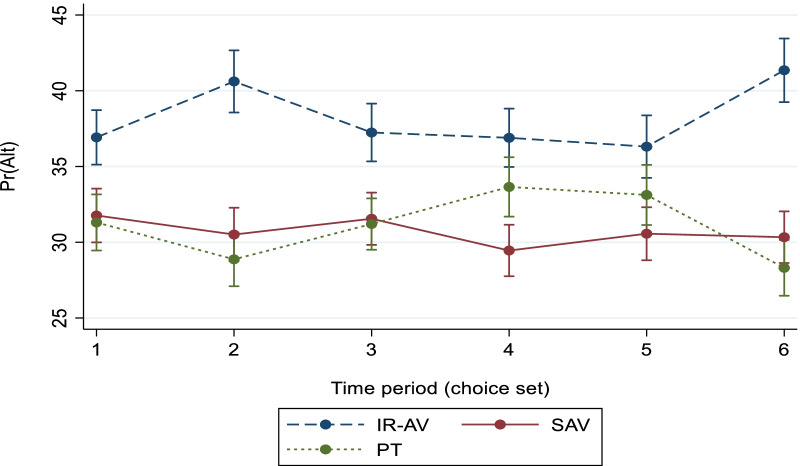
The predictive margins with 95% confidence level across the time period

The power of the developed model (i.e., ML model) is its ability to demonstrate the changes on the margins across the choice sets (i.e., time period), for example, the margins of the alternatives at a trip time across the choice sets (i.e., six choice sets are given for each individual in the SP survey) are varied, as shown in Fig. 7.

### The conditional marginal effects

**Fig. 8:**
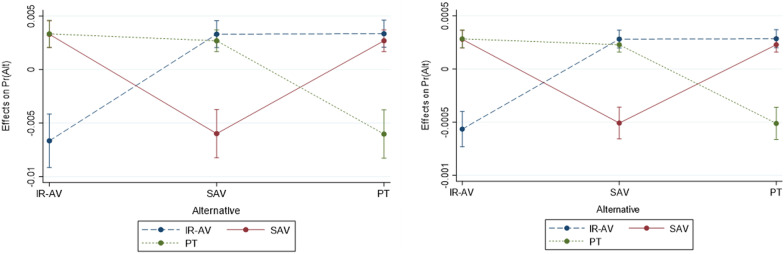
The conditional marginal effect of the trip time and trip cost (CI stands for confidence level)

The conditional average marginal effect of the travel time, travel cost, and main onboard activity is derived from Table 5. Figure 8 demonstrates that the marginal effect of the travel cost is different than that of the travel time. The graphs are used to understand the impact of modifying the travel time and the travel cost of each alternative on the probability of choosing a specific alternative. The graphs show the marginal utility of the trip time and trip cost, for example, the effect of changing the trip time of IR-AV on IR-AV is − 6.6e−3, on SAV is 5.9e−3, and on PT is 6e−3, while the effects of changing the trip cost of IR-AV on IR-AV is − 5.6e−4, on SAV is 5e−4, and on PT is 5.1e−4.

**Fig. 9:**
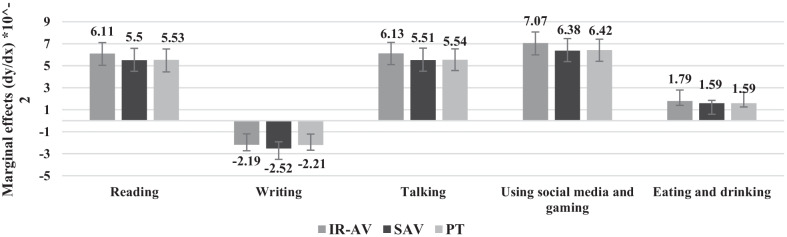
The marginal effects of main onboard activity on the predicted probability of IR-AV, SAV, and PT

The marginal effect of onboard activities on an alternative concerning IR-AV, SAV, and PT is shown in Fig. 9 and Table 6. Generally, it is shown that the difference between the modes within each activity category was not significant. The marginal effects are used to explain the variations in the predictive margin where different margins are obtained based on the availability of main activity on board of a transport mode. When reading activity is possible, the marginal effects on choosing IR-AV, SAV, and PT compared to doing nothing (i.e., others) increases by 0.0611, 0.0550, and 0.0553, respectively. The marginal effects on choosing IR-AV, SAV, and PT compared to doing nothing when writing activity is possible is − 0.0219, − 0.0252, and − 0.0221, respectively. When talking activity is possible, the marginal effects on choosing IR-AV, SAV, and PT compared to doing nothing increases by 0.0613, 0.0554, and 0.0551, respectively. When using social media and gaming activity is possible, the marginal effects on choosing IR-AV, SAV, and PT are the highest. The probability of an individual to choose AV when using social media and gaming activity is possible compared to doing nothing increases by 0.0707, 0.0638, and 0.0642, respectively. The marginal effects on choosing IR-AV, SAV, and PT compared to doing nothing is the lowest when the eating/drinking activity is possible.

**Table 6:** The marginal effects of main onboard activity on the predicted probability of IR-AV, SAV, and PT

	Reading	Writing	Talking	Social media and gaming	Eating and drinking
IR-AV	6.11	− 2.19	6.13	7.07	1.79
Standard errors	1.06	0.55	1.03	1.07	0.39
SAV	5.5	− 2.52	5.51	6.38	1.59
Standard errors	1.08	0.6	1.09	1.1	0.26
PT	5.53	− 2.21	5.54	6.42	1.59
Standard errors	1.09	0.47	0.98	1.01	0.34
Significance level (%)	99	85	99	99	95

Table 6 shows the marginal effects of main onboard activity on the predicted probability. The findings show significant results at confidence level of at least 95% except for writing activity which shows significant result at confidence level of 85%.

The results show that the marginal effect of main onboard activity on IR-AV is different than on SAV and on PT, while SAV and PT show solely a slight difference. To summarize, the expected probability of an individual to choose an alternative, decreases when writing onboard is available. This means that writing is not preferred by the travelers (i.e., a negative sign) when compared to other activities due to several reasons: the short time of the trip, the unwillingness of travelers to be involved in writing during traveling to their main trip purpose to avoid stress and to avoid missing the final station in case of PT, as well as not having the permission or access to their work in case their main trip is to go to work. Other onboard activities demonstrate a positive utility, which means that travelers convert part of their travel time to productive time based on the macroeconomic time allocation theory. The talking and using social media and gaming activities do not show significant differences across SAV and PT because they share almost the same environment on board excluding the potential onboard congestion. The onboard eating/drinking activity shows significant differences across the three transport modes, where the probability of choosing SAV and PT is lower than IR-AV when the eating/drinking activity is possible.

The result of the analysis shows that the preferences of travelers in urban areas, where the travel time is short, do not show a large difference on the utility of travelers across alternatives of similar environments, such as SAV and PT. Furthermore, it is noted that the travelers are passengers in case of the three alternatives with different environments, such as the companions on board of SAV and PT is possible, while in case of IR-AV, it is not available. Travelers are less likely to conduct writing activity onboard even in the age of automated driving. The travel and traveler characteristics affect the preferences of travelers onboard, such as job, age, and car ownership variables.

## Discussion

People’s travel behavior is studied through an ML model, where three different transport modes are included. The individuals’ preferences are assessed based on their main trip purposes in urban areas, where a trip is relatively short. Some characteristics of the trips and the travelers are collected to examine their impacts on the transport mode choice. The travel behavior when choosing a transport mode is determined based on the following three variables: trip time, trip cost, and main onboard activity possibility. The findings demonstrate the impact of those three travel variables on the probability of using a transport mode. Moreover, other variables are examined, where solely variables with reasonable significance are kept in the model, such as current regular transport mode, age, job, and car ownership.

The results of this research highlight that each onboard activity shows a different utility on choosing a transport mode as follows: writing activity demonstrates a negative utility on the transport mode choice compared to doing nothing. This concludes that travelers avoid writing on board during traveling to their main activities. The reasons are one or more of the followings: the lack of required tools on board, not sufficient privacy on board, insufficient trip time (i.e., short trip), permission for access to work files are not given, writing needs stationary activity, and travelers seem to be used to using social media rather than writing for a specific purpose, such as work email, making a shopping list, or planning leisure activities. Besides, it is demonstrated that to be involved in writing activity has the least negativity in case of IR-AV, and the highest is in case of SAV, as shown in Figs. 4, 5 and 6. The results of the model demonstrate that other onboard activities show positive utility on the potential transport mode choice compared to doing nothing. The highest positive utility is found when travelers use social media and gaming. This finding is partially consistent with one of the findings of Lee et al. [41], who demonstrate that using ICT tools is the most anticipated activity on board of AV. In their research, the authors do not differentiate between IR-AV and SAV.

Current study presents the margins of use based on the developed model as a whole and based on the transport mode variables. The highest margin is found in case of IR-AV, and the lowest is found in case of SAV. Furthermore, a little difference is found between the margins of SAV and PT. The impact of the trip time and trip cost on the probability of choosing a transport mode is obtained. The result demonstrates that an increment in trip time by 10% or a reduction in the trip cost by 10% in case of IR-AV keeps the highest probability for using IR-AV, as shown in Fig. 3. Thus, the probability of choosing IR-AV is predominant even if there are slight changes in the variables. Additionally, the largest probability is demonstrated in IR-AV where the changes on the availability of the main onboard activities is done, as shown in (see also Figs. 4, 5, and 6). It is found that IR-AV is the predominant transport mode across all onboard activities. The highest margin is obtained when the using social media and gaming activity is available (i.e., main activity), and the lowest is obtained when writing is available (i.e., main activity). Furthermore, the results highlight that the least margin is found in SAV with a slight difference on PT across all main onboard activities. Therefore, IR-AV is the predominant transport mode where travelers can find their preferences mostly fulfilled. Besides, SAV and PT show close values with a preference of SAV.

The model shows the margins of choosing each alternative. The sensitivity of margins using a certain alternative due to the change in one variable in the same alternative or in other alternatives as well as the change across the choice set can be derived from the model. The prediction of choosing an alternative per travel time and travel cost is presented, and the results show various values of predictions per time change and cost change. The conditional margin effects of modifying variables on the selection of a transport mode are discussed based on the trip time, the trip cost, and the main onboard activity variables. The change in trip time and in the trip cost is accompanied by different changes based on a transport mode, for example, the change in probability is found to be the highest in IR-AV. The results demonstrate that the probability of using IR-AV is different than on SAV and on PT when changing from doing nothing/others to each one of the onboard activities, as shown in Figs. 8 and 9.

Other traveler attributes show varied impacts on using a transport mode, for instance, based on the developed model, the probability of using SAV for people who are drivers is the lowest, and almost people with the highest potential to use PT are drivers. Thus, drivers are less likely to accept ridesharing to multitask during travel. Youngsters (i.e., 15–24 years) and the elderly (i.e., 65 + years) are more likely to use SAV and PT, respectively, than other groups. Elderly people are less likely to multitask than youngsters because the older generation faces difficulties in using ICT tools, as demonstrated by Nikitas et al. [50]. Furthermore, elderly people’s willingness to use IR-AV is the least due to their preferences, such as technophobia. Moreover, Nikitas et al. [50] state that elderly people are not in the labor force which emphasizes their fluctuated opinion on AV,they are not even sure whether they might witness its coming or not. Based on a confidence level of 95%, the results demonstrate that the age group of 25–54 are more likely to use SAV than other groups. Job types show different utilities in the potential choice of a transport mode. For example, full-time workers prefer using SAV and PT more than other groups. The car ownership variable demonstrates low probability when using PT or SAV.

The travel time and travel cost coefficients show negative impacts, which reflects the negative impact of travel on the travelers’ daily life. The travelers’ VOT is 870 HUF/hour (i.e., 2.64 Euro per hour). The willingness to pay for travel time savings per transport mode is derived from Fig. 8.

This study simulates one situation where travelers can only choose one main activity onboard due to the short trip time. In this case, a traveler is able to choose one activity as the main activity based on his/her preferences with the possibility to conduct other activities as secondary activities. The study is designed where a traveler interacts with the assigned random choice sets as a real case and answer accordingly. The study simulates the real facts, that a traveler uses a transport mode based on his preferences, such as possibility of conducting one main activity, possibility of conducting secondary activity/ies, using future technology (IR-AV, and SAV). The outputs of the study have fruitful results to the vehicles manufacturers, policy makers, and transport operators. Moreover, the IR-AV and SAV are not on the market yet, where a traveler experience is not obtained, this is a limitation of the study.

## Policy implications

The preferences of travelers onboard in the automated driving age are manifested. The travelers choose a transport mode based on their preferences. The probability of choosing a transport mode based on trip time, trip cost, main onboard activity, job, car ownership, and age variables is explained. The preferences vary across the alternatives, which shows that people differentiate between IR-AV, SAV, and PT when they want to be involved in onboard activities during traveling to the sites of their main activities in urban areas. Each onboard activity shows different utilities on each alternative as well as on the trip time and trip cost. It is noted from Fig. 9 that SAV and PT demonstrate more similar values because their in-vehicle environment is more similar once compared to IR-AV. Drivers and youngsters are more likely to use IR-AV over SAV and PT, while full-time workers are keen to use either PT or SAV over other job groups. The effect on the prediction of choosing the alternatives based on the available onboard activities is discussed, which suggests some policy implications, such as hardness in accepting ridesharing. Besides, IR-AV seems to be predominant in urban areas, which leads to an increase in congestion since the individual mode is preferred over SAV and PT by the travelers. Moreover, SAV is preferable over PT, which leads to negative impact on the PT and more vehicles on the road due to the potential shift from PT to SAV.

## Limitations

The limitations of this research include the geographic area of the study which consists of respondents from Hungary alone, as well as the research focuses exclusively on short trips in urban areas. Moreover, the sample does not represent the population because it is based on a selected group of people due to the sensitivity of the study. Elderly people are less likely to accept the participation in this kind of service due to their opinion on recent technology. Besides, the research includes solely the travelers’ main trips. Additionally, the findings are based on the participants’ ability to choose based on their preferences and their understanding of a situation where new technology is on the market rather than on their experience, for example, distinguishing between IR-AV and SAV. The choices are limited to three transport modes, which makes other users of non-motorized modes choose one of the alternatives. Other secondary activities are not covered in this study as well as including more than one activity as main activities in the DCE are not examined either. The result of this study is based on the PT of Budapest, which is well-developed; thus, the results may be positively influenced by the available infrastructure and the local travelers’ current travel behavior.

## Recommendations

The results of this study are applied in the urban areas and for the travelers’ main trips. Therefore, it is recommended to study other alternatives in the future and to analyze the obtained utilities of onboard activities with long distance trips outside urban areas. The presented onboard activities can be improved by proposing a combination of two or more activities, similarly to the study of Wardman et al. [69]. The impacts of sociodemographic and trip characteristics show varied results that can be enhanced by using a larger sample size with a diverse variety of respondents in terms of sociodemographic and trip characteristics. Moreover, studying the impact of ICT tools availability on board provided either by the travelers or by the operators (i.e., vehicle) on the transport choice is a potential for further research.

## Conclusion

The SP survey is applied to collect information on the traveler preferences and perspectives regarding their current transport modes and their preferences toward IR-AV, SAV, and PT. The survey includes participants with various categories in terms of job, age, income, gender, car ownership, and education. A sample size of 525 participants is collected and analyzed to develop a transport mode choice model based on the travel time, travel cost, and main onboard activity. The discrete choice modeling approach is applied, where the ML model is used to study the traveler preferences and the potential probabilities of choosing alternatives across the observed variables and individual responses. The developed model studies the added dis/utility of six onboard activities (i.e., reading, writing, talking, using social media and gaming, eating/drinking, and doing nothing) on transport mode choice. The onboard activities demonstrate various effects on the travel behavior across the alternatives. The study highlights the preferences of a group of people in transport mode choice. Moreover, the impacts of the trip time and trip cost changes on the travel behavior are analyzed. The results show that people are more likely to choose IR-AV over other modes, while choosing SAV has the lowest probability. Reading and using social media affect the choice of a transport mode positively to a greater extent than other activities. Writing activity is not preferred by travelers. The variations on the impact of onboard activities are demonstrated, such as reading, using social media, and gaming adds positive utility for travelers’ utilities, while writing adds negative utility for travelers. This research adds a contribution that emphasizes the importance of onboard activities during traveling to the main trip purpose in urban areas, such as the utility of each onboard activity is estimated when IR-AV, SAV, and PT are considered.

## Data Availability

The data are included in the manuscript and further information is provided upon request.
